# Supplementary material for: Multi‐dataset identification of innovative feature genes and molecular mechanisms in keratoconus
Source: J Cell Mol Med. 2024 Sep 19;28(18):e70079. doi: 10.1111/jcmm.70079 (PMC11412914; doi:10.1111/jcmm.70079)
Supplement: Supplementary file 2 — Material S2. [file JCMM-28-e70079-s002.zip › 7.GSEA analysis/4.MYRF/KEGG_ARRHYTHMOGENIC_RIGHT_VENTRICULAR_CARDIOMYOPATHY_ARVC.html]

Details for gene set KEGG\_ARRHYTHMOGENIC\_RIGHT\_VENTRICULAR\_CARDIOMYOPATHY\_ARVC[GSEA]

|  || Dataset | MYRF.group.cls#h\_versus\_l |
| Phenotype | group.cls#h\_versus\_l |
| Upregulated in class | l |
| GeneSet | KEGG\_ARRHYTHMOGENIC\_RIGHT\_VENTRICULAR\_CARDIOMYOPATHY\_ARVC |
| Enrichment Score (ES) | -0.52224195 |
| Normalized Enrichment Score (NES) | -1.6316286 |
| Nominal p-value | 0.01004016 |
| FDR q-value | 0.21474732 |
| FWER p-Value | 0.538 |
Table: GSEA Results Summary

  

Fig 1: Enrichment plot: KEGG\_ARRHYTHMOGENIC\_RIGHT\_VENTRICULAR\_CARDIOMYOPATHY\_ARVC      
 Profile of the Running ES Score & Positions of GeneSet Members on the Rank Ordered List

  

| SYMBOL | TITLE | RANK IN GENE LIST | RANK METRIC SCORE | RUNNING ES | CORE ENRICHMENT || 1 | CACNG8 | na | 755 | 0.506 | 0.0140 | No |
| 2 | CACNG5 | na | 1534 | 0.398 | 0.0197 | No |
| 3 | ITGA4 | na | 1618 | 0.389 | 0.0448 | No |
| 4 | DAG1 | na | 4906 | 0.225 | -0.0341 | No |
| 5 | CACNB1 | na | 5538 | 0.206 | -0.0378 | No |
| 6 | ITGA9 | na | 5848 | 0.197 | -0.0328 | No |
| 7 | EMD | na | 9064 | 0.115 | -0.1174 | No |
| 8 | ITGB7 | na | 9321 | 0.109 | -0.1171 | No |
| 9 | DSC2 | na | 9618 | 0.103 | -0.1183 | No |
| 10 | CACNB2 | na | 10834 | 0.081 | -0.1477 | No |
| 11 | CACNA2D3 | na | 11412 | 0.072 | -0.1592 | No |
| 12 | DES | na | 11440 | 0.071 | -0.1550 | No |
| 13 | CACNA1C | na | 11956 | 0.064 | -0.1653 | No |
| 14 | ITGB6 | na | 12239 | 0.060 | -0.1692 | No |
| 15 | DSP | na | 12258 | 0.060 | -0.1655 | No |
| 16 | ACTB | na | 12485 | 0.057 | -0.1680 | No |
| 17 | CACNA1D | na | 13802 | 0.040 | -0.2031 | No |
| 18 | ATP2A2 | na | 14195 | 0.036 | -0.2119 | No |
| 19 | DMD | na | 14406 | 0.033 | -0.2156 | No |
| 20 | CTNNA2 | na | 14571 | 0.032 | -0.2181 | No |
| 21 | ACTN4 | na | 14881 | 0.029 | -0.2250 | No |
| 22 | TCF7L2 | na | 15138 | 0.027 | -0.2305 | No |
| 23 | CTNNB1 | na | 15600 | 0.022 | -0.2422 | No |
| 24 | CACNG7 | na | 16278 | 0.015 | -0.2607 | No |
| 25 | CTNNA1 | na | 16494 | 0.013 | -0.2660 | No |
| 26 | CACNG1 | na | 17979 | -0.000 | -0.3088 | No |
| 27 | CACNG2 | na | 18631 | -0.007 | -0.3271 | No |
| 28 | ITGB4 | na | 19208 | -0.013 | -0.3429 | No |
| 29 | JUP | na | 19857 | -0.020 | -0.3601 | No |
| 30 | LMNA | na | 20403 | -0.027 | -0.3740 | No |
| 31 | GJA1 | na | 22120 | -0.047 | -0.4202 | No |
| 32 | ACTG1 | na | 22317 | -0.050 | -0.4223 | No |
| 33 | LAMA2 | na | 23159 | -0.062 | -0.4422 | No |
| 34 | ITGA3 | na | 23712 | -0.071 | -0.4531 | No |
| 35 | ITGB5 | na | 23730 | -0.071 | -0.4485 | No |
| 36 | ITGA2 | na | 25150 | -0.096 | -0.4827 | No |
| 37 | CTNNA3 | na | 25302 | -0.099 | -0.4801 | No |
| 38 | ITGA2B | na | 25913 | -0.111 | -0.4898 | No |
| 39 | CACNB3 | na | 26225 | -0.118 | -0.4905 | No |
| 40 | ITGAV | na | 26862 | -0.132 | -0.4995 | No |
| 41 | SGCA | na | 27650 | -0.152 | -0.5115 | Yes |
| 42 | TCF7L1 | na | 27666 | -0.152 | -0.5012 | Yes |
| 43 | ACTN3 | na | 27890 | -0.157 | -0.4965 | Yes |
| 44 | ACTN1 | na | 27985 | -0.160 | -0.4879 | Yes |
| 45 | ITGA11 | na | 28180 | -0.166 | -0.4818 | Yes |
| 46 | SGCG | na | 28859 | -0.187 | -0.4882 | Yes |
| 47 | CACNA1F | na | 28862 | -0.187 | -0.4750 | Yes |
| 48 | SGCD | na | 29214 | -0.197 | -0.4712 | Yes |
| 49 | ITGA7 | na | 29295 | -0.200 | -0.4594 | Yes |
| 50 | ITGA8 | na | 29346 | -0.201 | -0.4466 | Yes |
| 51 | ITGA6 | na | 29414 | -0.203 | -0.4342 | Yes |
| 52 | CACNG6 | na | 29465 | -0.205 | -0.4212 | Yes |
| 53 | ITGB3 | na | 29529 | -0.207 | -0.4083 | Yes |
| 54 | ITGB1 | na | 29873 | -0.219 | -0.4027 | Yes |
| 55 | TCF7 | na | 30107 | -0.227 | -0.3934 | Yes |
| 56 | ACTN2 | na | 30149 | -0.229 | -0.3784 | Yes |
| 57 | RYR2 | na | 30209 | -0.231 | -0.3638 | Yes |
| 58 | PKP2 | na | 30420 | -0.239 | -0.3529 | Yes |
| 59 | CDH2 | na | 30517 | -0.243 | -0.3385 | Yes |
| 60 | ITGB8 | na | 30931 | -0.260 | -0.3321 | Yes |
| 61 | DSG2 | na | 31792 | -0.306 | -0.3352 | Yes |
| 62 | CACNG4 | na | 31907 | -0.312 | -0.3165 | Yes |
| 63 | CACNA2D2 | na | 32005 | -0.317 | -0.2969 | Yes |
| 64 | ITGA10 | na | 32096 | -0.323 | -0.2766 | Yes |
| 65 | SGCB | na | 32475 | -0.349 | -0.2629 | Yes |
| 66 | CACNA2D4 | na | 32488 | -0.350 | -0.2385 | Yes |
| 67 | CACNA1S | na | 33196 | -0.406 | -0.2302 | Yes |
| 68 | SLC8A1 | na | 33227 | -0.410 | -0.2020 | Yes |
| 69 | LEF1 | na | 33408 | -0.432 | -0.1767 | Yes |
| 70 | ITGA1 | na | 33768 | -0.482 | -0.1530 | Yes |
| 71 | CACNB4 | na | 34188 | -0.569 | -0.1249 | Yes |
| 72 | CACNG3 | na | 34232 | -0.580 | -0.0851 | Yes |
| 73 | ITGA5 | na | 34505 | -0.679 | -0.0450 | Yes |
| 74 | CACNA2D1 | na | 34582 | -0.726 | 0.0042 | Yes |
Table: GSEA details [plain text format]

  

Fig 2: KEGG\_ARRHYTHMOGENIC\_RIGHT\_VENTRICULAR\_CARDIOMYOPATHY\_ARVC      
 Blue-Pink O' Gram in the Space of the Analyzed GeneSet

  

Fig 3: KEGG\_ARRHYTHMOGENIC\_RIGHT\_VENTRICULAR\_CARDIOMYOPATHY\_ARVC: Random ES distribution      
 Gene set null distribution of ES for **KEGG\_ARRHYTHMOGENIC\_RIGHT\_VENTRICULAR\_CARDIOMYOPATHY\_ARVC**
